# Large ungulates will be present in most of Japan by 2050 owing to natural expansion and human population shrinkage

**DOI:** 10.1038/s41598-026-38177-4

**Published:** 2026-02-06

**Authors:** Takahiro Morosawa, Hayato Iijima, Tomonori Kawamoto, Takahisa Kanno, Ryota Araki, Teruki Oka

**Affiliations:** 1 Japan Wildlife Research Center, 3-3-7, Kotobashi, Sumida-ku, Tokyo, 130-8606 Japan; 2 Forestry and Forest Products Research Institute, Matsunosato 1, Tsukuba, Ibaraki 305-8687 Japan; 3 Wildlife Management Center, Tokyo University of Agriculture and Technology, 3-5-8, Saiwaicho, Fuchu, Tokyo 183-8509 Japan

**Keywords:** Climate, Dispersal ability, Distribution expansion, Sika deer, Wild boar, Biogeography, Climate-change ecology, Macroecology

## Abstract

The aims of this study were to elucidate factors contributing to the expansion of the distributions of sika deer and wild boar in Japan and to predict the expansion of their distributions by 2025, 2050, and 2100. A predictive model was constructed using information on species distribution collected by the Ministry of the Environment in 1978, 2003 and 2014, days of snow cover, forested and road areas, elevation, human population, and distance to the nearest occupied cell as covariates to calculate the probability of distribution change. Factors contributing to distribution expansion were elucidated and distribution expansion was predicted. Distance to the nearest occupied cell had the strongest influence on distribution expansion, followed by the inherent ability of each species to expand its distribution. For sika deer, human population had a negative effect and forest area and number of days of snow cover have positively affected. For wild boar, forest area, snow days and population had high importance. Predictions of future distribution showed that both species will be distributed nationwidely by 2050.

**Supplementary Information:**

The online version contains supplementary material available at 10.1038/s41598-026-38177-4.

## Introduction

Large ungulates have both positive and negative effects on human life. They offer services of hunting^1^, venison^2^, and medicine^3^ and disservices of the irreversible alteration of ecosystems by over-browsing^4–6^, economic damage in forestry and agriculture^7^, collisions with vehicles^8^, and the transmission of zoonoses^9^ and of disease vectors such as ticks^10,11^. Planning for future human well-being depends on the prediction of population expansion of large ungulates and related factors.

The distribution of large ungulates has continued to expand in recent years, especially in developed countries, and it is urgent to understand why^12–15^. Factors related to their expansion include climate change^16^, land use change^17,18^, and hunting^15,19–21^. Because global climate change is expected to continue, it may accelerate the expansion of their distribution^16,22^. The relationship between climate and distribution has been related to snow^23–26^, and it is expected that as snowfall decreases with climate change, species distributions will expand into more northern and heavier-snowfall regions. In addition, human populations are shrinking in developed countries, so associated changes in land use, such as abandonment of land^17^ and reduced hunting pressure^17,27^, may accelerate expansion. Therefore, predicting distribution expansion and planning responses in regions where the distributions of large ungulates are expected to expand is important.

To predict the expansion of large ungulates, researchers have used species distribution models (SDMs^23,28^ to assess environmental factors influencing expansion. SDMs are widely used for predicting the expansion of distribution and explaining the relative importance of environmental factors related to expansion^29^. Environmental factors in newly occupied areas can differ from those in original habitats, yet animals adapt to the new resources^30^. Thus, spatial information is also an important aspect of distribution prediction and including this information should lead to higher predictive power of SDMs.

As both the likelihood of distribution expansion and the similarity of environmental factors depend on distance, it is important to consider spatial information in relation to both. Incorporating spatial information such as distance from the current distribution into models while taking environmental factors into account will improve the realism of predictions. In addition, while adding temporal information allows for better predictions, the computational load is even greater. Therefore, when temporal dynamics are estimated in large-scale spatial predictions, ways to reduce the computational load must be considered.

In Japan, large-scale land use changes have occurred since the period of rapid economic growth with expanded afforestation, population growth, and urbanization began in the 1960s^31^. Under these changes, the populations of large ungulates such as sika deer and wild boar have recovered under national protection policies^24,32^. Both species were distributed throughout Honshu in the prehistoric Jomon period, but their distributions had been shrinking since the Meiji period (1868–1912)^33^. They expanded again during the period of expanded afforestation after World War II^15^. Since then, the distributions of both species have continued to increase, sika deer by 2.7 times in area and wild boar by 1.9 times from 1978 to 2018^34^. In 2018, both species were reported to have expanded into the northern Tohoku and Hokuriku regions, where they had not lived since the end of World War II owing to heavy snowfall^34^. With such dynamic changes in landscape and wildlife distribution and its wide latitudinal gradient, Japan is a suitable region in which to evaluate factors associated with changes in wildlife distribution. Recently, Baek et al. (2025)^35^ reported that climate change and the increase in abandoned farmland have contributed to the expansion of the distribution of large mammals in Japan. On the other hand, the dispersal capacity that large ungulates have may contribute more strongly than environmental factors.

The main aim of this study was to clarify factors important for population expansion of large ungulates using hierarchical models. In particular, we tested whether the mobility possessed by sika deer and wild boars, rather than the physical environment or climatic factors, contributed to their distribution expansion. Finally, we also predicted the distribution of both sika deer and wild boar up to 2100 using related factors.

## Results

### Factors contributing to distribution expansion

While no significant coefficient was found for the probability of continued distribution (φ), significant coefficients were estimated for the probability of new establishment (γ). For new establishment (γ), the distance to the nearest occupied cells had the strongest negative effect on colonization (γ) for both species (Tables 1 and 2). For sika deer, forest area and snow days had positive effects, whereas human population, elevation, and distance to the nearest occupied cell had negative effects (Table 1). For wild boar, forest area had positive effects, whereas human population, elevation and distance to the nearest occupied cell had negative effects (Table 2). However, the coefficient for the number of snow days was positive under Representative Concentration Pathways (RCP) 2.6, but negative under RCP 8.5.

**Table 1:** Estimated coefficients of predictive variables in Sika deer. Median and 95% credit intervals were given.

Variable	φ		γ	
	RCP2.6	RCP8.5	RCP2.6	RCP8.5
Intercept	5.19 (3.16 – 8.64)	5.11 (3.17 – 7.93)	−5.84 (−6.44 - −5.28)	−5.86 (−6.34 - −5.36)
Population	−0.54 (−1.36 - −0.02)	−0.55 (−1.33 - −0.02)	−0.26 (−0.38 - −0.14)	−0.26 (−0.37 - −0.14)
Forest area	1.32 (0.64 – 2.16)	1.31 (0.65 – 2.11)	0.24 (0.19 – 0.29)	0.23 (0.19 – 0.29)
Elevation	4.65 (1.92 – 9.08)	4.52 (1.97 – 8.85)	−0.15 (−0.20 - −0.11)	−0.15 (−0.20 - −0.11)
Snow days	1.77 (1.41 – 2.23)	1.75 (1.40 – 2.22)	0.23 (0.18 – 0.27)	0.22 (0.18 – 0.27)
Road area	0.05 (−0.39 – 0.44)	0.05 (−0.39 – 0.43)	0.05 (−0.02 – 0.12)	0.05 (−0.02 – 0.13)
Distance from occupied cell			−9.78 (−5.46 - −4.84)	−9.81 (−10.55 - −9.05)

**Table 2:** Estimated coefficients of predictive variables in wild boar. Median and 95% credit intervals were given.

Variable	φ		Γ	
	RCP2.6	RCP8.5	RCP2.6	RCP8.5
Intercept	176.47 (78.2 – 317.88)	182.3 (89.11 – 327.94)	−2.84 (−3.01 - −2.67)	−10.04 (−10.84 - −9.44)
Population	−39.68 (−101.45 – 111.81)	33.30 (−51.05 – 184.43)	−0.31 (−0.41 - −0.21)	−0.23 (−0.30 - −0.17)
Forest area	13.59 (−77.07 – 83.33)	−32.51 (−140.42 – 58.90)	0.33 (0.29 – 0.36)	0.76 (0.72 – 0.82)
Elevation	18.84 (−60.76 – 142.75)	40.28 (−34.00 – 161.65)	−0.16 (−0.20 - −0.13)	−0.23 (−0.29 - −0.18)
Snow days	24.7 (−45.22 – 114.46)	−9.23 (−112.83 – 99.37)	0.25 (0.22 – 0.29)	−0.30 (−0.40 - −0.22)
Road area	0.02 (−22.41 – 121.96)	38.50 (−25.98 – 183.13)	0.03 (−0.03 – 0.09)	0.04 (−0.00 – 0.08)
Distance from occupied cell			−5.15 (−5.42 - −4.88)	−15.67 (−16.9 - −14.72)

Comparing between RCP 2.6 and RCP 8.5, the coefficient of new establishment (γ) was larger at RCP 8.5 for both sika deer and wild boar (Tables 1 and 2). While other coefficients were not different for sika deer, the coefficient of snow days was positive at RCP 2.6 but negative at RCP 8.5 for wild boar.

### Distribution prediction

**Fig. 1:**
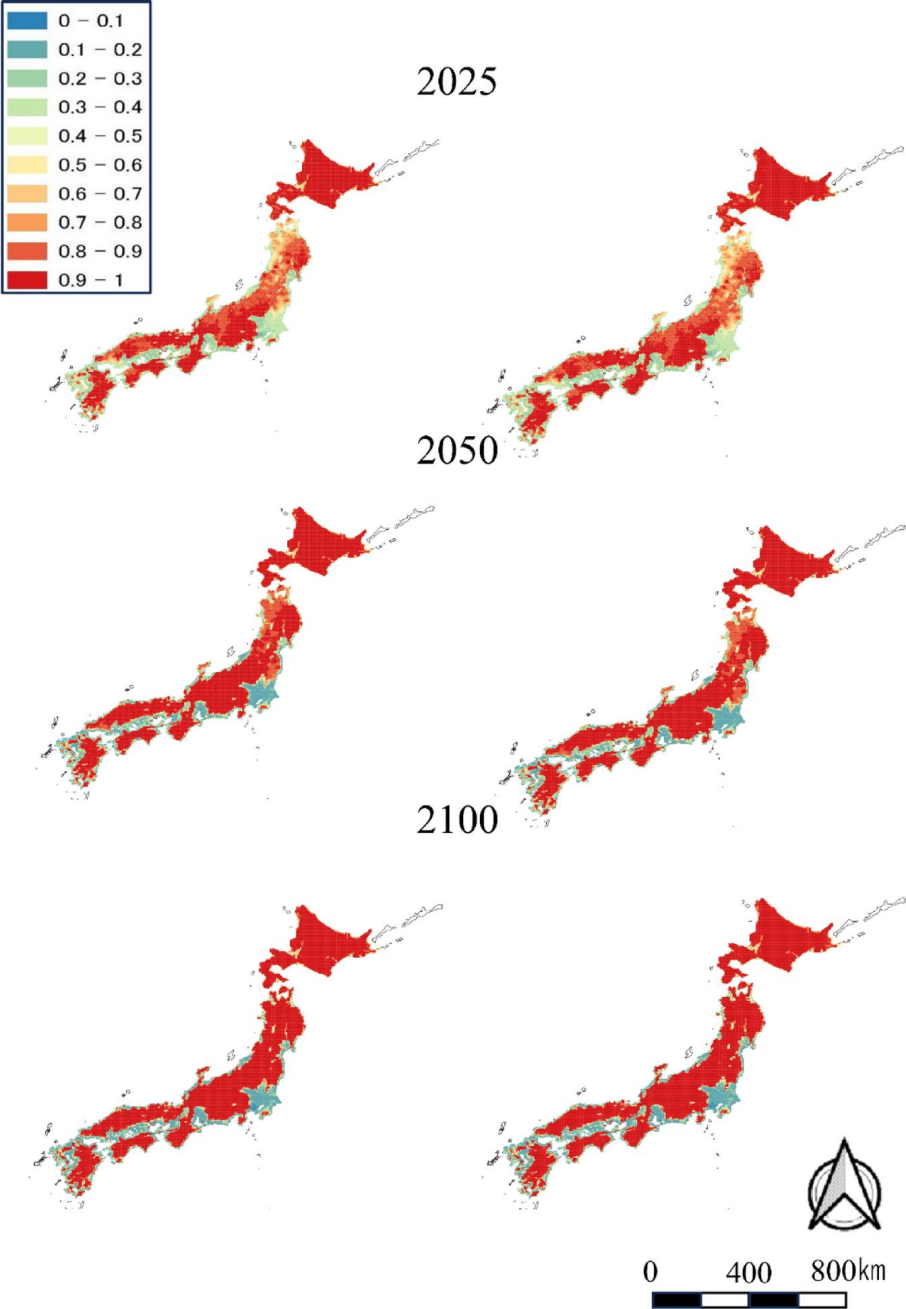
Predicted probabilities of deer distributions in 2025, 2050, and 2100 under the RCP2.6 (left column) and RCP8.5 (right column) climate change scenarios. The maps were created by using QGIS ver. 3.40.8 and Microsoft Power Point office 2019 based on administrative boundary data from the National Land Numerical Information (https://nlftp.mlit.go.jp/ksj/index.html) available under CC-BY-4.0.

**Fig. 2:**
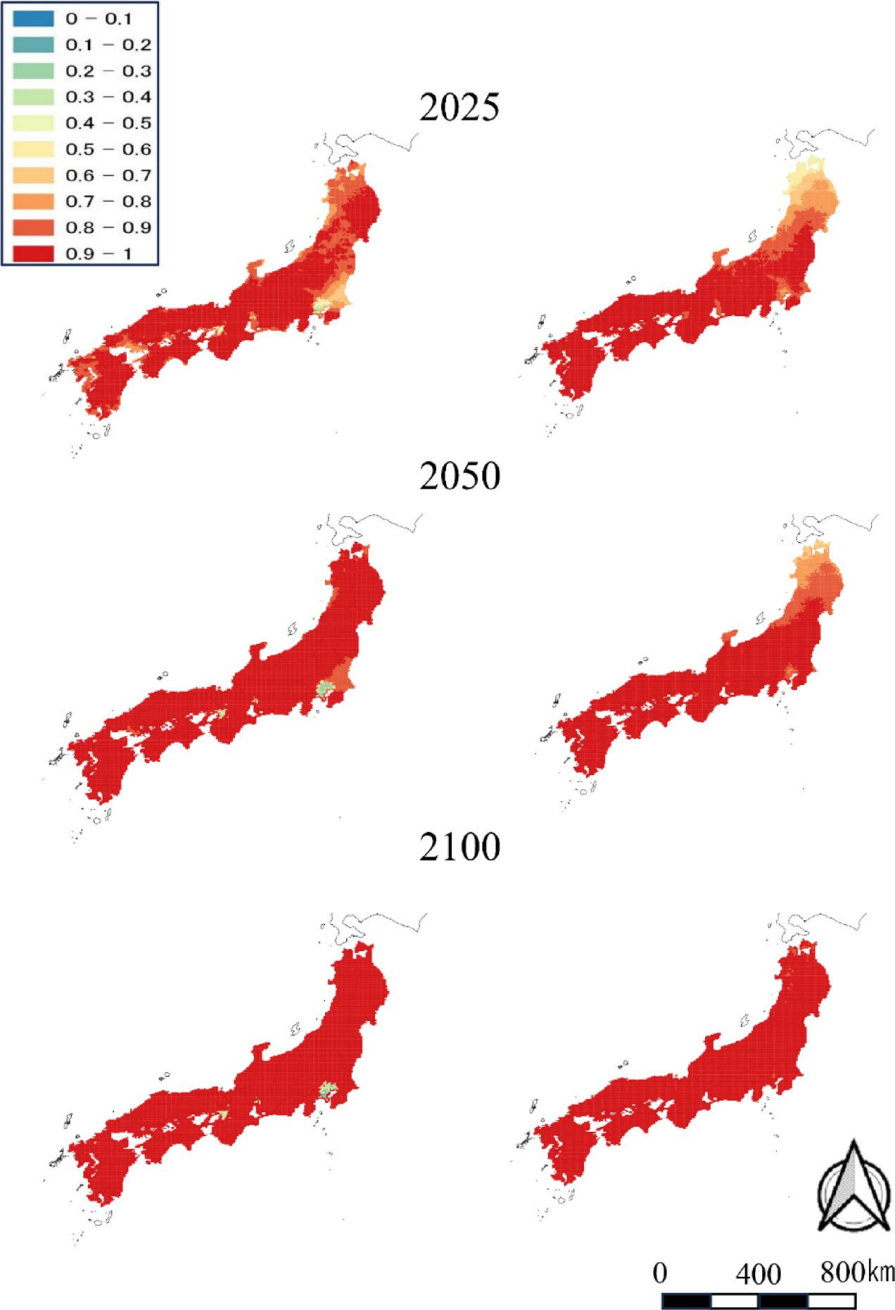
Predicted probabilities of wild boar distributions in 2025, 2050, and 2100 under the RCP2.6 (left column) and RCP8.5 (right column) climate change scenarios. The maps were created by using QGIS ver. 3.40.8 and Microsoft Power Point office 2019 based on administrative boundary data from the National Land Numerical Information (https://nlftp.mlit.go.jp/ksj/index.html) available under CC-BY-4.0.

In the distribution prediction models for sika deer and wild boar under the RC P2.6 scenario, the correct response rate within the time range for which distribution data were available (i.e., 1978 to 2014) was 100%, confirming the model’s high predictive power. The predictions show that sika deer will be present in wide range of Japan except Kanto region and low-elevation coastal areas by 2050 (Fig. 1). Wild boar will expand their distribution throughout Honshu region, Shikoku region and Kyusyu region by 2050 (Fig. 2). Under RCP 2.6 and RCP 8.5, the results of both species are very similar between scenarios.

## Discussion

We identified the relative importance of factors that contributed to the expansion of sika deer and wild boar populations between 1978 and 2014 and their differences between the species (Tables 1 and 2). By incorporating distance to the nearest occupied cell as spatial information, we examined the effects of climate, human population, and land use on the expansion of wild boar and sika deer distributions, improving both the spatial scale and reliability of predictions compared with previous studies. The importance of distance to the nearest occupied cell is also indicated by the fact that the coefficient of snow days for both species is greater for the model that did not use distance than for the model that used distance (Supplement Table 1). Additionally, by comparing the species, we obtained new findings.

As previous studies about climate change repeatedly showed, snowfall may be one of the important factors defining the distribution of large ungulates^16,23,24,36,37^. Although snow days affected the population expansion of both species, its effect was positive on sika deer. Our results suggest that the distribution of sika deer is likely to expand in the future in areas where snow depth is still high. Recent reports indicate that coniferous forests, which tolerate snow cover, function as wintering grounds for sika deer^38,39^, which can be seen in large numbers there even in areas with heavy snowfall, where sika deer have not historically been seen^24^, supporting our results. The opposite coefficient of snowfall days under RCP 2.6 and RCP 8.5 for wild boars is thought to be related to snow depth and the short leg length of wild boars. Similar to sika deer, wild boar also showed more likely new establishment in areas with heavier snowfall under the RCP 2.6 scenario. On the other hand, comparing RCP 2.6 and RCP 8.5, the coefficient of distance to the nearest occupied cell and forest area were larger for RCP 8.5. Under RCP 8.5, as climate change progresses, the impact of snow days was thought to become relatively smaller, while the effects of distance to the nearest grid cell and forest area become larger. Deep snow depth limits the expansion of wild boar distribution, but under RCP 8.5, where climate change progresses and snow depth decreases, it is thought to promote the expansion of wild boar distribution. Wild boar have been expanding their distribution into in polar regions recently^24^. Vetter et al. (2020)^40^ reported that winter temperature increases associated with climate change affect the growth rate and structure of wild boar populations, and such mechanisms may also be relevant to the distribution expansion outcome of this study. The slightly higher distribution probability in 2050 under RCP 8.5 indicates that climate change may accelerate the distribution expansion of wild boar in particular. These results suggest that increasing climate change will accelerate distributional expansion similar to results of Baek et al. (2025)^35^.

With regard to land use, forest area had a positive effect, likely because forests represent relevant migration routes^37,42^ and feeding areas^41,43^ for both species. Especially on the Sea of Japan side of the country and in the Tohoku region, where distribution has been expanding in recent years^34^, forest area may serve as dispersal pathways to expand distribution.

Human population had a relatively high negative effect on distribution expansion. Sika deer distribution is expected to be restricted in urban areas^43^, particularly where hunting pressure is high^44^. On the other hand, behavioural flexibility allows wild boar to exploit human-dominated landscapes^45^, supporting our results. Thus, the influence of areas with greater human presence and potential conflict may depend on species.

Regarding factors related to new distributions, as previously mentioned, correlations were observed with several factors. On the other hand, no significant results were found for factors associated with continuing distributions. Once both species are established, they are unlikely to disappear unless they are subjected to extremely intense hunting or find themselves in other extraordinary conditions. In fact, from 1978 to 2003, only 1.7% and 6.1% of grid cells became absent in each species, respectively. Therefore, we conclude that no significant factors influencing φ could be identified. However, φ was estimated solely from a single transition period from 1978 to 2003, and the 2014 presence only data may have influenced the estimation results. Accordingly, the estimation results for φ are considered less robust than those for γ.

We predicted distributions in 2025, 2050, and 2100 from data collected in 1978, 2003 and 2014. The distributions of both species are still expanding, mainly on the Sea of Japan side of the country and in the Tohoku region, but our results expand the distributions almost nationwide by 2050. However, a difference was observed between sika deer and wild boars. While the distribution of sika deer did not expand in 2050 and 2100 in populated areas such as the Kanto region, the distribution of wild boars tended to expand even in urban areas. Similarly, Ohashi et al. (2016)^16^ included human population trends as an explanatory variable and made their prediction in consideration of the influence of human population changes on the distribution of sika deer and wild boar.

In considering the process of range expansion in this study, it is important to compare the historical distribution changes of both species. Sika deer was distributed throughout Japan during the Jomon period and remained nationwide during the Edo period. Subsequently, their range declined around 1950 due to intense hunting pressure^33^. Regarding population size, Iijima et al. (2023)^15^, who estimated effective population size using genetic information and revealed that the largest population size within the past 100,000 years was seen in two regions: Hokkaido and Hyogo Prefecture. Furthermore, over the past 100 years, a recovery in population size has been observed, similar to the distribution pattern. Although past population data for wild boar are unavailable, their distribution is reported to have followed a similar trajectory to that of sika deer^33^. While factors like snowfall and predators such as wolves likely constrained distribution, hunting pressure appears to have been the primary limiting factor for both species.

Our prediction results take climate change and other factors into account. However, limitations are that factors not considered may influence the results, potentially introducing uncertainty into the predictions. Examples of factors contributing to uncertainty include the classical swine fever (CSF), the predictive accuracy of the model, the uncertainty of the factors used for future projections, and hunting pressure. Regarding CSF, it has begun to spread among wild boar in Japan recently^46^, and an unexpected outbreak of infectious disease in sika deer cannot be ruled out. In addition, the Covid-19 epidemic has altered human behaviour. As for the model’s accuracy, the width of the confidence interval can also be interpreted as indicating uncertainty. The climate factors and land use employed for future projections are themselves model-predicted values. Making estimates based on these estimated values may further increase the uncertainty. Regarding hunting pressure, while it is expected to decrease in the future due to population decline, policy changes could potentially increase hunting pressure through management hunting as proposed by Iijima (in press)^47^. Therefore, in the medium to long term, changes in hunting pressure are quite likely to influence the distribution changes of both species.

Our predictions show that both sika deer and wild boar will be distributed wide range of Japan by 2050 and 2100. In 2016, Ohashi et al.^16^ projected that sika deer will be distributed in about 70% of the country by 2103, but our results suggest a faster expansion. Additionally, our study presents the first results for wild boar in Japan. Our study provides projections of future wild boar expansion in Japan, representing a useful tool to support management strategies aimed at mitigating impacts in newly colonized areas.

Our results indicate that the distribution of sika deer and wild boar will expand due to possible future changes in land use, human population dynamics, climate change, and other factors. Ongoing human population decline in Japan may further accelerate the expansion of both species.

Major countermeasures are population management by hunting and prevention of damage. Hunting pressure affects the distribution of large ungulates^19–21^. Thus, it may be possible to delay the expansion of distribution through hunting. Traditionally, hunting pressure has depended on local hunters, and strategic use has not been attempted in Japan. Our results will make it possible to examine, for example, where hunting pressure can be most effectively applied to delay expansion. In addition, since we clarified factors that are positively related to distribution expansion, it is possible to use our results to identify where hunting pressure should be intensively applied. Fences are set up after damage has occurred, but through the use of the predicted distributions, they can be set up in advance to prevent damage. In addition, our results can be used to prepare budgets in advance and to study damage control methods. Therefore, efficient and effective application of hunting pressure and damage control based on distribution prediction will become necessary in Japan.

## Materials and methods

### Data set of sika deer and wild boar distribution

**Fig. 3:**
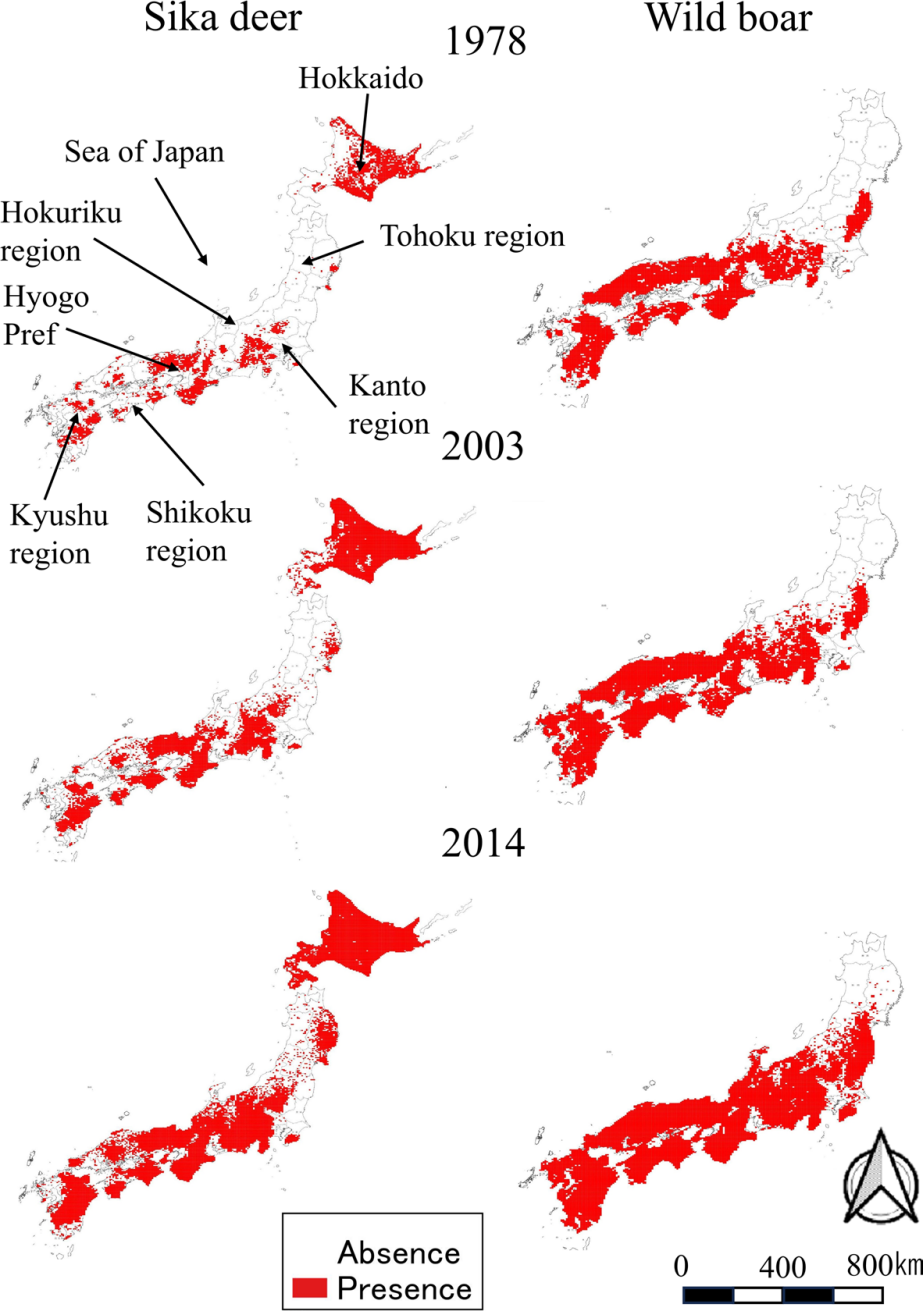
Presence data of sika deer (left column) and wild boar (right column). The maps were created by using QGIS ver. 3.40.8 and Microsoft Power Point office 2019 based on administrative boundary data from the National Land Numerical Information (https://nlftp.mlit.go.jp/ksj/index.html) available under CC-BY-4.0.

We used data collected by the Ministry of the Environment’s Natural Environment Survey in 1978 and 2003 from wildlife keepers and local government to map the distribution of sika deer and wild boar (Fig. 3, http://gis.biodic.go.jp/webgis/?_ga=2.193090862.2011052859.1653966617-587115043.1623632179.1653966617.1623632179.1653966617.1623632179). Data for 2014 were collected as areas where the distributions had expanded since 2003 as reported by local government hearing. Additionally, when reliable information such as captures is available, the distribution is added as an expansion since 2003. So, in 2014, only data on the presence of grid cells with expanded distributions from 2003 were collected, while data was not collected in a way that explicitly asked for the absence data in hearings (Fig. 3). Data for 2014 were provided by the Office of Wildlife Management, Ministry of the Environment. All data were collected and used on a 5-km × 5-km scale. Sika deer were distributed throughout Japan in Hokkaido, Honshu, Shikoku, and Kyushu, while wild boars were distributed in all regions except Hokkaido. Sika deer was distributed 3,890 cells in 1978, 7,184 cells in 2003, and 10,226 cells in 2014. Wild boars were distributed 5,067 cells in 1978, 6,509 cells in 2003, and 8,772 cells in 2014.

### Explanatory variables

#### Physical environmental variables

Average elevation, land use, and road area in each grid cell were drawn from national land numerical information (https://nlftp.mlit.go.jp/ksj/, 1-km × 1-km grid cell). The elevations recorded in 2009 were used for all years and averaged 1-km × 1-km grid cell data including in each 5-km × 5-km grid cell. Land use data in 1976, 2006, and 2015, which approximate the years of animal data, were also drawn from national land numerical information, from which areas of forest, agricultural land, paddy fields, water, and building land were extracted. Road area data in 1978, 2002, and 2010 were similarly drawn. In this dataset, land use is categorized for each 1-km × 1-km grid cell. Therefore, the area was calculated by summing the areas of land use categories within each 5-km x 5-km to create a data set. Road area of the 1-km x 1-km grid cells was also summed up with the 5-km x 5-km grid cells.

#### Climate variables

We used data on temperature and total rainfall from the meteorological stations of the Japan Meteorological Agency (JMA; https://www.data.jma.go.jp/obd/stats/etrn/) and calculated the averages. Although the JMA has about 1,300 meteorological stations nationwide, there are some areas where data are not available. Therefore, we interpolated missing values by spatial complementation of the values of other meteorological stations by Delaunay triangulation on a 5-km × 5-km scale. We used data on the deepest snow depth and the number of snow days provided by Ohashi and Kominami^16^. Furthermore, since there was more than 10 years interval between each set of distribution data, we assumed independence across time periods and did not consider temporal correlations among climate factors in this study.

#### Human population

We used national census data (1 km × 1 km; https://www.e-stat.go.jp/) from 1980, 2005, and 2015. The data of 1-km x 1-km grid cells contained in a 5-km x 5-km grid cells was summed up.

#### Distance from grid cells in which ungulates are present

Distances from grid cells in which sika deer and wild boar are present were calculated for 1978, 2003, and 2014 in GIS (ArcGIS v. 10.5). We calculated the center of each cell from the 5-km grid data and calculated the distances between centers. In the case of cells in which a species is present, the distance is 0 km; for a cell in which the species is absent, the distance to the nearest occupied cell was calculated.

#### Data for future prediction

For future prediction, we used the above physical environment data, meteorological data, human population, distance to the nearest occupied cell, and the 2009 elevation data. For land use, road area, and human population, we used the results predicted by the National Institute for Environmental Studies’ “S8” project (https://www.nies.go.jp/s8_project/). In the “S8” project’s predictions were made in multiple scenarios, but values with median sea level were used in this study. For meteorological data, we used predictions made by the Ministry of the Environment with the MRI-NHRCM20 regional climate model at a 20-km scale, using the same values for each 5-km × 5-km cell within each larger cell, and the median SST2 as the sea surface temperature. Ten-year average weather data were used in consideration of the uncertainty of the predictions. In predicting future distributions, we used the RCP 2.6 climate change scenario, which has the lowest temperature rise, and RCP 8.5, which has the highest temperature rise, to compare the effects of climate change.

### Model construction

In constructing a model for the expansion of the species’ distribution, we first considered a model in which the distribution probability is explained only by population, land use and climatic factors. However, strong spatial autocorrelation in spatially close environments may lead to incorrect predictions if spatial autocorrelation is not taken into account^48^. For this reason, it was necessary to consider spatial autocorrelation as well. However, it was difficult to do so here because of the high computational load due to the large number of cells (16,023) and the prediction of time-series distribution expansion. Therefore, we opted to add the distance to the nearest occupied cell as an explanatory variable^49^. This method is appropriate when the factors causing spatial autocorrelation are biologically clear^50^. In particular, mammals have strong mobility, and dispersal processes and their spatial parameters^39,41^ are considered more important than the environment when predicting distribution. As the purpose of this study was to evaluate how the ability of a species to expand its distribution affects the actual distribution, we consider using this method an appropriate method.

The use of statistical models to estimate potential habitat is common in predicting distributions^51^. However, problems can include not considering changes over time and overestimating the environmental effect of a yet-unoccupied or newly occupied area^52^. Considering temporal change as here allows us to evaluate the selectivity of the environment by taking into account the process of dispersion, making predictions more realistic.

Mammals in particular have a strong ability to disperse, and the process of dispersal, or its spatial parameters, is considered to be more important than environment in predicting distribution. Thus, unless certain environmental factors strongly limit distribution, mobility or dispersal ability contributes more strongly than environment to the expansion of the distribution.

To construct the distribution expansion model, we used a hierarchical model based explicitly on an observation model that explains the probability of distribution in each cell in terms of environmental factors and distance to the nearest occupied cell, and a process model that determines the presence or absence of distribution in each cell^53^. The model is based on both the probability that presence/absence at a grid cell will continue unchanged and the probability that a grid cell will be newly occupied. We constructed a distribution prediction model using the species distribution data and environmental factor data prepared above. First, we tested the correlations between the factors by Spearman’s rank correlation, and for those with ρ ≥ 0.6, we used either one. We found a strong correlation between the number of snow days and temperature; snow cover has been shown to influence the distribution of both species^54,55^. We selected the number of snow days.

The dynamics of a target species is expressed by the process model as:

$$z_{{i,k}} = z_{{i,k - 1}} \times \varphi + \left( {1 - z_{{i,k - 1}} } \right) \times \gamma$$

where *z*_*i, k*_ is the probability of the presence of a target species in the *i*th cell in the *k*th year; φ is the probability of survival; and γ is the probability of establishment. The equation predicts the presence/absence of the species in each cell from the sum of these probabilities. As the initial distribution (i.e.; Z_1,k_), we assumed a uniform Bernoulli distribution with expected values of 0 to 1, and used random numbers to correspond to the distribution data.

We expanded the process model by incorporating covariates into φ and γ. The value of φ and γ in each cell will be affected by the environment, land use, meteorological factors, human population, and distance to the nearest occupied cell, so we expressed it as:

$$\varphi _{i} = 1 - \exp \left( { - \exp \left( \begin{gathered} \alpha + \beta 1 \times HumanPopulation_{i} + \beta 2 \times ForestArea_{i} \hfill \\ + \beta 3 \times Elevation_{i} + \beta 4 \times SnowDays_{i} + \beta 5 \times RoadArea_{i} \hfill \\ \end{gathered} \right) \times SurveyInterval_{k} } \right)$$

$$\gamma _{{i,k}} = 1 - \exp \left( { - \exp \left( \begin{gathered} \alpha + \beta 1 \times HumanPopulation_{{i,k}} \hfill \\ + \beta 2 \times ForestArea_{{i,k}} + \beta 3 \times Elevation_{i} \hfill \\ + \beta 4 \times SnowDays_{{i,k}} + \beta 5 \times RoadArea_{{i,k}} \hfill \\ + \beta 6 \times Dis\tan ceNearestcell_{{i,k}} \hfill \\ \end{gathered} \right) \times SurveyInterval_{k} } \right)$$

For φ, distance is excluded from the factor because of the probability that it continues to be distributed in a grid cell. In addition, data on absence were collected only in 1978 and 2003, and only presence data were collected thereafter, so φ was estimated only from changes in presence/absence in 1978 and 2003. For 2014, only the newly expanded distribution data, for which the distribution was reliably confirmed, were used. The models assumed that capture pressure, which affects the distribution of both species, does not change significantly from the period for which distribution data are available, although this was not explicitly stated in the future projections. The analysis was conducted separately for sika deer and wild boar.

Using the complementary log–log function as above allows us consider the effect of the difference between survey intervals on the presence/absence of wildlife. The function takes into account the variation in intervals between survey years, which are used as an offset term. In addition, as the interval of the year for which the distribution data is obtained is not equal, we used the interval of the year predicted by including the predicted interval of the year as the change in the distribution per unit time. As the coefficients of each factor, we set a normal distribution (0, 1000) as the prior distribution. In addition, since the large number of cells to which factor data were only partially applied stymied the estimation, we used the method of estimating the factor itself from the data and substituting it. The factor was calculated as a normal distribution (expected value of factor, variance of factor), and its variance as a hyperparameter with a uniform distribution (0, 100). All explanatory variables were standardized before analysis, so the relative importance of the coefficients could be evaluated from their magnitude by comparing the estimates. A Markov Chain Monte Carlo model was run with a burn-in of 50,000 steps, a calculation 200,000 steps, and thinning of 15. We judged that the calculated $\:\widehat{R}$ converged below 1.1^56^. The calculation was performed by the JAGS v. 4.3.0^57^ in R ver. 4.02^58^.

## Supplementary Information

Below is the link to the electronic supplementary material.

Supplementary Material 1

## Data Availability

All datasets are available from the corresponding author on reasonable request. The code used is available from the corresponding author on reasonable request.
