# Supplementary material for: Large ungulates will be present in most of Japan by 2050 owing to natural expansion and human population shrinkage
Source: Sci Rep. 2026 Feb 6;16:7550. doi: 10.1038/s41598-026-38177-4 (PMC12932835; doi:10.1038/s41598-026-38177-4)
Supplement: Supplementary file 1 — Supplementary Material 1 [file 41598_2026_38177_MOESM1_ESM.docx]

# Appendix

Table 1 Estimated coefficients (median and 95 % credible intervals) of predictive variables in each species model without distance.

| Variable | Sika deer | | Wilde boar | |
| --- | --- | --- | --- | --- |
|  | phi | gamma | phi | gamma |
| Intercept | -1.19  (-1.33 - -1.09) | 14.24  (9.08 - 21.02) | -1.25  (-1.59 - -1.19) | 86.29  (-1.82 - 146.1) |
| Population | -1.76  (-2.26 - -1.35) | -0.11  (-0.57 - 0.14) | -0.38  (-0.47 - -0.07) | 6.09  (-2.1 - 40.28) |
| Forest area | 0.39 (0.33 - 0.45) | 2.82  (1.76 - 4.58) | 1.17  (0.88 - 1.26) | -33.32  (-70.1 - 4.74) |
| Elevation | -0.09  (-0.14 - -0.04) | 11.72  (6.45 – 20.00) | 0.82  (0.49 - 0.89) | -22.95  (-46.86 - 5.03) |
| Snow days | -0.01  (-0.07 - 0.05) | 2.99  (2.24 - 4.07) | -2.44  (-2.58 - -1.69) | 71.3  (-11.02 - 130.74) |
| Road area | 0.15  (0.04 - 0.24) | 0.41  (0.15 - 0.94) | 0.13  (0.04 - 0.18) | 0.39  (-11.36 - 11.95) |
